# Authors' reply to: Commentary to: Lymphocytic UV autofluorescence: A novel ultraviolet-induced fluorescence dermoscopy finding in lichen nitidus—A series of 2 cases

**DOI:** 10.1016/j.jdcr.2025.12.007

**Published:** 2026-01-02

**Authors:** Varun H, Adarshlata Singh, Meenakshi Chandak, Bhushan Madke, Prerit Sharma, Vangala Naga Nitya

**Affiliations:** Department of Dermatology, Venereology and Leprosy, Datta Meghe Institute of Higher Education and Research, Jawaharlal Nehru Medical College, Wardha, Maharashtra, India

**Keywords:** autofluorescence, dermatoscopy, dermoscopy, lichen nitidus, lymphocytes, novel, ultraviolet-induced fluorescence dermoscopy (UVFD)

*To the Editor:* We thank Pietkiewicz et al for their valuable and insightful comments and for contributing to the discussion on UV-induced fluorescence dermoscopy (UVFD) in lichen nitidus (LN). We appreciate their alternative hypothesis; however, several points merit clarification.

LN features a thinned, stretched, hypomelanotic epidermis overlying a compact lymphocytic infiltrate, which permits greater UV penetration than in other inflammatory dermatoses such as lichen planus, eczema, or folliculotropic mycosis fungoides, where hyperkeratosis and acanthosis limit UV transmission due to reflection and the Stokes shift phenomenon.^1^, ^2^, ^3^ This structural difference may influence the detectability of fluorescence arising from deeper infiltrates.

The brown dots/shadows in LN are well-recognized dermoscopic correlates of the lymphocytic ball, and in our cases, fluorescence occurred almost exclusively in papules containing these brown dots.^4^, ^5^, ^6^ Papules lacking brown dots showed little to no fluorescence—or at least not the brilliant, sharply circumscribed fluorescence seen in brown-dot lesions. Biopsies were taken specifically from the fluorescent papules, supporting a direct topographic correlation.

Although lymphocytic fluorescence is typically white-blue and bilirubin tends to fluoresce yellow-green, this distinction is only a weak supportive point, as fluorescence spectra can overlap and color alone cannot reliably differentiate fluorophores (Fig 1). More importantly, the structures we observed were smooth-surfaced, intrapapular foci, not surface crusts (Fig 2). If the brown dots represented excoriations with sero-hemorrhagic crusts, they would be expected to appear dark on UVFD, as hemoglobin absorbs UV rather than fluoresces—contrary to our findings.^7^^,^^8^ Case 1 also lacked pruritus, reducing the likelihood of excoriations.

**Fig 1:**
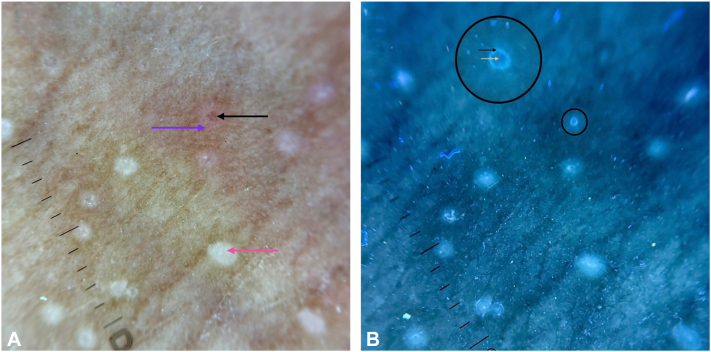
**A,** Cross-polarized dermoscopy (DermLite DL5, coupled with iPhone 16 camera) showing an LN papule with a central excoriation containing a sero-hemorrhagic crust (*black arrow*). Surrounding inflammatory erythema secondary to scratching is visible (*purple arrow*). A newly formed LN papule without a developed brown dot is also noted (*red arrow*). **B,** UVFD (DermLite DL5, Wood-Mode, coupled with iPhone 16 camera) of the same field. The small black circle marks the region corresponding to the probable sero-hemorrhagic crust. The larger black circle highlights the same area in magnified view. Under UVFD, this focus demonstrates darkening, most likely due to UV absorption by hemoglobin (*yellow arrow*). Adjacent greenish-white fluorescence is seen (*purple arrow*), a finding most compatible with bilirubin-related fluorescence. *LN*, Lichen nitidus; *UVFD*, UV-induced fluorescence dermoscopy.

**Fig 2:**
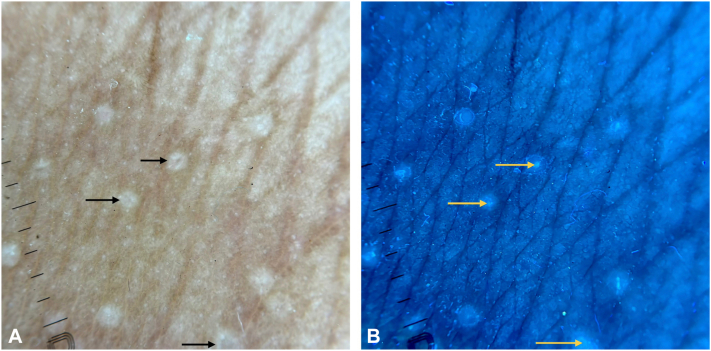
**A,** Cross-polarized dermoscopy (DermLite DL5, coupled with iPhone 16 camera) showing multiple LN papules with central brown dots/shadows (*black arrows*), with no surrounding erythema. **B,** UVFD (DermLite DL5, Wood Mode, coupled with iPhone 16 camera) of the same field showing bright fluorescence—the lymphocyte UV autofluorescence sign—in the exact regions corresponding to the brown dots/shadows (*yellow arrows*). Notably, there is no UV-induced darkening, making sero-hemorrhagic crusts unlikely as the source of these structures. *LN*, Lichen nitidus; *UVFD*, UV-induced fluorescence dermoscopy.

We agree that further studies are needed to refine UVFD interpretation. We thank the authors again for their thoughtful contribution.

Sincerely,

H et al.

## Conflicts of interest

None disclosed.
